# The past and present of serum aminotransferases and the future of liver injury biomarkers

**DOI:** 10.17179/excli2016-800

**Published:** 2016-12-15

**Authors:** Mitchell R. McGill

**Affiliations:** 1 Div. of Laboratory and Genomic Medicine, Dept. of Pathology and Immunology; 2 Dept. of Medicine, Washington University School of Medicine, St. Louis, MO, USA

**Keywords:** liver injury, liver disease, diagnostics, biomarkers, mechanistic biomarkers

## Abstract

Laboratory testing is important in the diagnosis and monitoring of liver injury and disease. Current liver tests include plasma markers of injury (e.g. aminotransferases, γ-glutamyl transferase, and alkaline phosphatase), markers of function (e.g. prothrombin time, bilirubin), viral hepatitis serologies, and markers of proliferation (e.g. α-fetoprotein). Among the injury markers, the alanine and aspartate aminotransferases (ALT and AST, respectively) are the most commonly used. However, interpretation of ALT and AST plasma levels can be complicated. Furthermore, both have poor prognostic utility in acute liver injury and liver failure. New biomarkers of liver injury are rapidly being developed, and the US Food and Drug Administration the European Medicines Agency have recently expressed support for use of some of these biomarkers in drug trials. The purpose of this paper is to review the history of liver biomarkers, to summarize mechanisms and interpretation of ALT and AST elevation in plasma in liver injury (particularly acute liver injury), and to discuss emerging liver injury biomarkers that may complement or even replace ALT and AST in the future.

## Introduction

Laboratory testing is important in the diagnosis of liver disease and the differentiation of etiology. The current battery of liver tests can roughly be divided in three: 1) indicators of liver function, 2) indicators of liver injury and 3) viral hepatitis serologies. Tests of liver function comprise coagulation tests (e.g. prothrombin time, INR), serum bilirubin and serum protein (total or albumin), while liver injury tests include serum alanine aminotransferase (ALT), aspartate aminotransferase (AST), alkaline phosphatase (ALP), γ-glutamyl transferase (GGT), lactate dehydrogenase (LDH) and sometimes glutamate dehydrogenase (GLDH). Alpha-fetoprotein (AFP) stands alone in a fourth category as a biomarker of hepatocyte proliferation (e.g. liver development, hepatocellular carcinoma, liver regeneration). Among the liver injury markers, ALT and AST are probably the most commonly used in both clinical diagnosis and research involving liver damage. However, there is some confusion about the proper use and interpretation of these aminotransferases (Senior, 2012[60]). Furthermore, emerging data have highlighted the limitations of serum aminotransferases for early detection of injury and for prediction of patient outcome. 

Recent years have seen the proliferation of liver injury biomarkers, many with the potential to replace or at least complement serum aminotransferases for certain applications (Amacher et al., 2013[1]; McGill and Jaeschke, 2014[43]). Considerable progress has been made in the development of novel mechanistic biomarkers (McGill and Jaeschke, 2014[43]), markers of inflammation (McGill and Jaeschke, 2014[43]), extracellular RNA-based biomarkers (Starkey Lewis et al., 2012[63]; Amacher et al., 2013[1]; McGill and Jaeschke, 2015[44]), and various others (Davern et al., 2006[14]; Craig et al., 2013[13]; Prima et al., 2013[55]; McGill et al., 2014[42][45][47]; Weerasinghe et al., 2014[69]). It has been suggested that one or more of these markers will replace or supplement current biomarkers for some purposes in the near future. In fact, the US Food and Drug Administration (FDA) and the European Medicines Agency (EMA) both expressed support for several organizations interested in developing new drug-induced liver injury biomarkers, including the Safer and Faster Evidence-based Translation network and the Drug-Induced Liver Injury Network. Thus, it seems appropriate to review what is known about the biology and clinical utility of the aminotransferases, what we know about the mechanisms of serum aminotransferase elevation and the proper interpretation of serum levels, and what the future may hold for biomarkers in the diagnosis and monitoring of liver injury. 

## Reactions Catalyzed by the Aminotransferases

**Figure 1:**
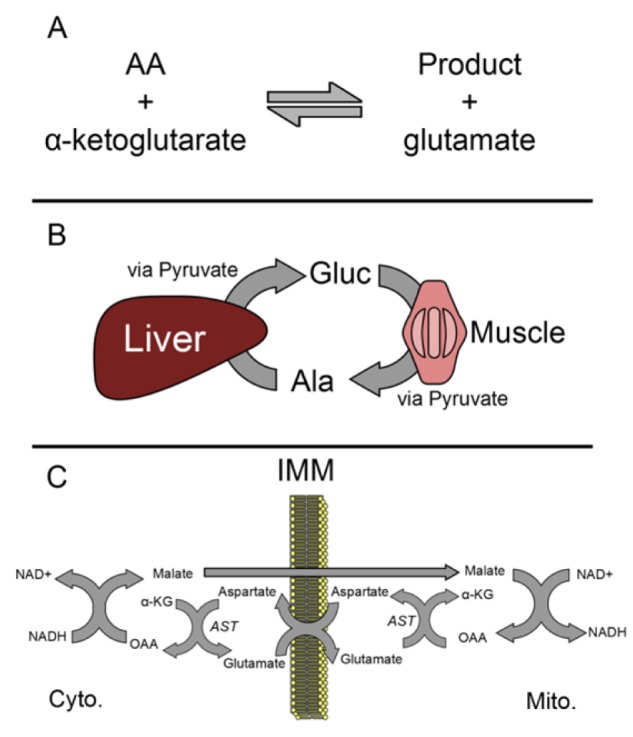
Functions of ALT and AST. (A) Both alanine and aspartate aminotransferases (ALT and AST, respectively) catalyze the conversion of alpha-ketoglutarate (a-KG) and an amino acid to glutamate and another product. In the case of ALT, the amino acid and product are alanine and pyruvate. In the case of AST, the amino acid and product are aspartate and oxaloacetate (OAA). (B) The glucose-alanine cycle. (C) The malate-aspartate shuttle. IMM, inner mitochondrial membrane.

Both ALT and AST catalyze the transfer of an amino group from an amino acid to α-ketoglutarate. The amino acids are L-alanine and L-aspartate and the reaction products are L-glutamate and either pyruvate or oxaloacetate, respectively (Figure 1A(Fig. 1)). The overall effect is exchange of an amino group and a keto group. Pyridoxal 5'-phosphate (PLP; vitamin B6 derivative) serves as a coenzyme in both reactions. It is important to note that these reactions can be reversed. Because the reactants and products are important for numerous cell processes, both ALT and AST have diverse physiological functions aside from their obvious roles in amino acid metabolism. For example, they are also important for energy homeostasis. Arguably, the most important role of ALT is in the alanine-glucose cycle (Figure 1B(Fig. 1)). In muscle, ALT converts pyruvate to the amino acid alanine using an amino group from glutamate. Alanine enters circulation and is taken up by the liver, where ALT in hepatocytes can convert it back to pyruvate which can be used to make glucose. This system is especially important for glucose regulation during stressful conditions such as fasting or vigorous exercise. It has also been suggested that the mitochondrial isoform of ALT is particularly important in gluconeogenesis in some cases (McCommis et al., 2015[41]). The most important physiological function of AST may be maintenance of the NAD+/NADH ratio within cells. AST is a critical partner in the malate-aspartate shuttle, which oxidizes NADH in the cytosol and reduces NAD+ in the mitochondrial matrix to facilitate glycolysis and electron transport, respectively (Figure 1C(Fig. 1)). 

## Brief History of Clinical Liver Tests

Prior to the advent of liver enzyme measurement, assessment of liver health was limited to clinical examination (e.g. jaundice, abdominal pain) and laborious tests of liver function. For example, direct bilirubin could be measured as early as 1913 using the van den Bergh reaction. The clearance of dyes such as bromosulphthalein, (Rosenthal and White, 1925[59]) as well as formation of metabolites of compounds such as benzoic acid (Quick, 1936[57]), provided an indication of the ability of the liver to metabolize and excrete xenobiotics. The galactose tolerance test assessed the ability of the liver to convert galactose to glycogen (Bauer, 1906[7]; Shay et al., 1931[61]). Flocculation of samples after mixing with specific reagents, such as cephalin-cholesterol complexes prepared from sheep brain (Hanger, 1939[21]), could be examined to measure changes in serum protein composition caused by liver dysfunction. Coagulation time could also be measured. Unfortunately, most of these tests were relatively time-consuming, or were not broadly applicable to many liver diseases. Furthermore, the large functional capacity of the liver and its ability to regenerate mean that many of these tests would not be expected to give abnormal results except in cases of very severe or prolonged liver disease. Thus, sensitivity was probably limited for most of these tests.

Although ALP had been measured in serum in various diseases as early as 1930 (Kay, 1930[31]), changes in other liver enzyme levels, including aminotransferase levels, in serum from patients with liver injury were not described until much later. At the time, it was generally thought that intracellular enzymes were anchored to cell organelles and could not be released even after plasma membrane damage (Ettre and Zlatkis, 1979[18]). It took the boldness of a private practice physician with only limited laboratory training and a medical student assistant to pursue the possibility. Karmen et al. (1955[30]) were the first to report elevated ALT and AST in serum during liver injury. They observed aminotransferase increases in a single patient with acute hepatitis and two others with cirrhosis in the early 1950s (Karmen et al., 1955[30]). The authors were specifically interested in investigating the possibility of using these enzymes as markers of myocardial infarction, but found that they could be elevated in other disease states as well. Around the same time, De Ritis et al. (1955[15]) demonstrated an increase in both enzymes in serum from viral hepatitis patients. Interestingly, Karmen and Wróblewski (1955[30]) may have been the first to use the term “biochemical biopsy,” which was used in reference to the aminotransferases (Wróblewski, 1958[73]).

Initially, the measurement of aminotransferase activity could also be time-consuming. In the study by Karmen et al. (1955[30]), paper chromatography was used to separate glutamate produced by the reaction of either enzyme with α-ketoglutarate and alanine or aspartate. The glutamate spots were then cut from the paper and the glutamate was extracted and measured with a colorimetric test. In an appendix to their groundbreaking study, Karmen et al. (1955[30]) introduced a coupled enzyme reaction for AST measurement that is the basis for many modern assays. A very similar test was later developed for ALT (Wróblewski and LaDue, 1956[75]). In the former case, the oxaloacetate produced by AST was reduced to malate by malate dehydrogenase, consuming NADH in the process. In the latter case, pyruvate produced by ALT was further reduced by LDH, also consuming NADH. The depletion of NADH over time can be monitored by loss of absorbance at 340 nm and these data can be used to calculate activity. Coupled reactions dramatically reduced the amount of time needed to measure these enzymes (Ettre and Zlatkis, 1979[18]). 

**Figure 2:**
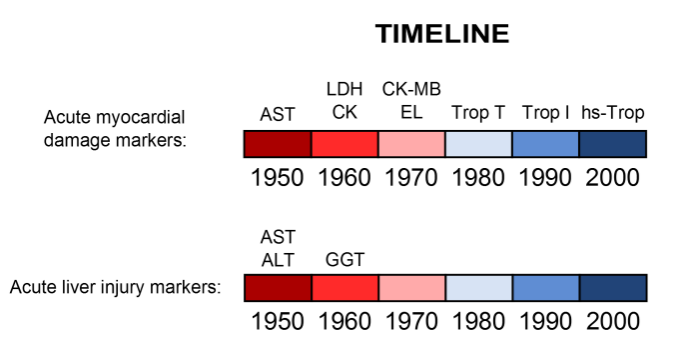
Timeline of biomarker development for both acute myocardial injury and liver injury (1950 - 2010). AST, aspartate aminotransferase ALT, alanine aminotransferase LDH, lactate dehydrogenase GGT, gamma-glutamyl transpeptidase CK, creatine kinase CK-MB, creatine kinase-MB (myocardial isoform) EL, electrophoresis for LDH and CK Trop, troponin Hs-Trop, high sensitivity troponin

Measurement of transaminases was soon adopted in clinical laboratories. Although follow-up experiments revealed that AST was consistently elevated in patients with liver disease (Wróblewski and LaDue, 1955[74]; Jervis et al., 1956[26]), it was found that ALT is a better indicator of liver damage because, unlike AST, its activity is much greater in liver than in muscle (LaDue and Wroblewski, 1956[34]). Overall, the specificity of ALT for the liver and availability of a relatively fast method to measure it made it appealing for clinical use. Unfortunately, while markers of injury in other organs, such as heart and kidney, seem to be constantly evolving, aside from the introduction of GGT as a marker of liver disease in the 1960s (Szczeklik et al. 1961[65]) there has been very little change in biomarkers of liver injury in clinical use since the introduction of AST and ALT, in contrast to biomarkers for other forms of tissue injury including acute myocardial damage (Figure 2(Fig. 2)).

## Mechanisms and Interpretation of ALT/AST Elevation

It is generally thought that aminotransferase elevations are due to cell damage with plasma membrane disruption. This is supported by the finding that serum ALT levels are initially low after treatment with inducers of hepatocyte apoptosis, but increase later in the course of injury. Apoptotic cell death can be thought of as cell implosion; a controlled process with minimal release of proteins into the extracellular space. However, oncotic necrosis, which can be thought of as cell explosion, can occur in some cells secondarily after induction of apoptosis. The hallmarks of apoptosis (e.g. cell shrinkage, chromatin condensation, apoptotic body formation) are clearly observable in the liver in hepatocyte apoptosis models during the early phase of injury when there is no significant elevation in serum ALT (Leist et al., 1995[37]; Lawson et al., 1998[35]; Bajt et al., 2000[6]). The serum ALT activity dramatically increases during the later phase, when secondary necrosis occurs either as a result of direct hepatocyte damage or inflammation (Leist et al., 1995[37]; Lawson et al., 1998[35]; Bajt et al., 2000[6]).

Although plasma membrane damage and protein leakage is probably the most common reason for elevated serum ALT, there is evidence that other mechanisms can be involved (Table 1(Tab. 1)) (References in Table 1: Oncotic necrosis: Leist et al., 1995[37]; Lawson et al., 1998[35]; Bajt et al., 2000[6]; Membrane blebbing: Lemasters et al., 1981[38]; Kamiike et al., 1989[29]; Gores et al., 1990[20]; Increased expression: Pappas et al., 1980[50]; 1986[51]; 1989[52]; Aubert et al., 2012[5]; Macroenzymes: Konttinen et al., 1978[32]; Kajita et al., 1978[28]; Moriyama et al., 1990[49]; Briani et al., 2003[10]). It has been suggested that membrane blebs containing cytosolic components form during ischemia-reperfusion injury (IRI) of the liver and that these protrusions can rupture to release aminotransferases without cell death (Lemasters et al., 1981[38]; Kamiike et al., 1989[29]; Gores et al., 1990[20]). This was based in part on evidence from microscopy (Lemasters et al., 1981[38]) and on the finding that the cytosolic isoform of AST is released early in IRI, while the mitochondrial isoform does not increase in serum until later and correlates better with the extent of liver necrosis (Kamiike et al., 1989[29]). 

Another contributor to increased serum aminotransferase levels in some cases is induction of expression. It has been known for some time that microsomal enzyme-inducing drugs, including alcohol, can increase tissue expression and serum levels of GGT (Whitfield, 2001[70]). Experimental work has also revealed that ALT and AST activity are increased in livers of mice treated with carbon tetrachloride (CCl_4_), suggesting an increase in expression (Pappas, 1980[50], 1986[51]). Furthermore, post-treatment with the protein synthesis inhibitor cyclohexamide was shown to prevent this aminotransferase induction and significantly reduce serum levels of both enzymes (Pappas, 1986[51], 1989[52]). Importantly, cyclohexamide did not increase survival, demonstrating that the reduced aminotransferase levels were not simply a result of protection against CCl_4_ (Pappas, 1986[51]; 1989[52]). It was suggested that the increase in ALT and AST expression after CCl_4_ treatment could be partially explained by liver cell regeneration (Pappas, 1989[52]), but little evidence for that was presented. Overall, these findings support the idea that even large increases in serum levels of aminotransferases can be due, in part, to induction of expression. 

At least two mechanisms have been proposed for regulation of ALT expression. It has been known for decades that some patients taking fibrates have asymptomatic increases in serum ALT. Fibrates are peroxisome-proliferator-activated receptor-α (PPARα) agonists. This knowledge led to the hypothesis that PPARα regulates expression of ALT and AST, which was supported by the finding that treatment with fenofibrate induced expression of the cytosolic isoforms of both enzymes in human hepatoma cells (Edgar et al., 1998[17]). Interestingly, fenofibrate reduced expression of the cytosolic aminotransferases in mice and this effect was abrogated by PPARα deficiency (Edgar et al., 1998[17]). The latter finding supported the importance of PPARα in the expression of aminotransferases, while demonstrating a clear species difference in response to PPARα agonists. It was later shown that the cytosolic and mitochondrial isoforms of ALT (ALT1 and ALT2, respectively) are the products of two different genes (*GPT1* and *GPT2*) in both mice and humans (Sohocki et al., 1997[62]; Yang et al., 2002[76]; Jadhao et al., 2004[23]). *GPT1* is located on chromosome 8 while *GPT2* is on chromosome 16 (Sohocki et al., 1997[62]; Yang et al., 2002[76]). The cytosolic and mitochondrial isoforms of AST are also encoded by different genes (Pol et al., 1989[54]). GOT1 is located on chromosome 10, while GOT2 is on chromosome 16 and possibly also encoded in part on chromosomes 1 and 12. ALT1 is now known to be the dominant isoform of ALT in the liver (Lindblom et al., 2007[39]). Consistent with earlier work, further studies revealed that PPARα specifically controls expression of the *GPT1* gene (Thulin et al., 2008[66]). Fenofibrate treatment induced expression of ALT and increased binding of PPARα to the *GPT1* promoter in cultured human hepatocytes (Thulin et al., 2008[66]). Furthermore, deletion of the PPAR binding site in the promoter reduced fenofibrate-induced expression of *GPT1* (Thulin et al., 2008[66]). Altogether, there is strong evidence that PPARα plays a role in regulation of both ALT and AST levels, particularly ALT1. Other mechanisms seem to regulate *GPT2* expression, such as the PI3K-ATF4 axis (Hao et al., 2016[22]).

Recent work has shown that expression of ALT and AST can also be controlled by IRE1α/c-Jun signaling (Josekutty et al., 2013[27]). It was found that treatment with an inhibitor of the microsomal triglyceride transfer protein (MTP) increased levels of ALT1 and AST1 in both lysates and medium from Huh-7 cells, and knockdown of either IRE1α or c-Jun prevented these increases (Josekutty et al., 2013[27]). It is clear from these data that increased expression of ALT and AST genes can contribute to elevated serum levels. This may partially explain the wide variation in serum aminotransferase activities observed in humans during liver injury and the poor correlations of serum aminotransferases with extent of liver necrosis and patient outcome (Björnsson et al., 2006[9]; Antoine et al., 2012[3]; McGill et al., 2014[47]). Interestingly, it has been known for some time that various nutritional factors, such as protein intake, can affect aminotransferase levels (Rosen et al., 1959[58]). Obesity and steatosis have also been shown to cause a minor induction of ALT2 in the liver (Jadhao et al., 2004[23]; Aubert et al., 2012[5]). With increased obesity rates in humans, it is tempting to speculate that this phenomenon also contributes to the variation in serum ALT in liver injury patients. 

Overall, although cell death and plasma membrane damage are likely the dominant causes of serum aminotransferase elevations, other mechanisms can clearly influence the results. The actual mechanisms of release in the case of asymptomatic ALT or AST increases have not been well-studied. Conceivably, extracellular vesicles, like microvesicles and exosomes, or even protein secretion, could be involved. Furthermore, although it is usually assumed that baseline levels of serum aminotransferases are due to normal turnover of hepatocytes, it also possible that other mechanisms play a role. 

It should be noted that elevations in serum aminotransferase activities don't always involve increased release or expression. Complexes of serum enzymes with immunoglobulins or other proteins can also lead to moderately increased levels. Such “macroenzymes” can protect the serum enzymes from degradation, prolonging their half-lives and allowing them to accumulate to high concentrations. In this way, ALT and AST can be elevated even with normal release. A number of cases of aminotransferase macroenzymes have been described in the literature (Konttinen et al., 1978[32]; Kajita et al., 1978[28]; Briani et al., 2003[10]). Macroenzymes should be considered in cases of otherwise asymptomatic ALT or AST elevations in serum, especially if only one of the two is increased. One study found that approximately 13 % of cases of AST elevation without concomitant ALT increase are due to macroAST (Moriyama et al., 1990[49]).

## The Future of Liver Injury Biomarkers

There has been tremendous growth in interest in the development of new biomarkers of liver injury over the last decade. The three main drivers of this have been 1) the need during early drug trials for sensitive non-invasive biomarkers to identify new drugs that have the potential to cause idiosyncratic hepatotoxicity in a larger population, 2) the need for biomarkers to predict outcome in the clinical setting in which serious liver injury has already occurred, and 3) a desire for non-invasive biomarkers that are useful for translation of pathophysiological mechanisms from rodents to humans. The most common model used for identification of new biomarkers for all three purposes is the acetaminophen (APAP) overdose model. Although the injury caused by APAP is not idiosyncratic and its utility for the first purpose may be somewhat questionable, the APAP model is both experimentally convenient and clinically relevant. APAP hepatotoxicity can be induced in mice with a single large dose, and has a rapid disease course. In addition, APAP overdose is a common cause of acute liver failure (ALF) and ALF-related deaths in humans (Lee, 2008[36]) and it is easier to obtain clinical specimens from patients with liver injury caused by APAP than by other drugs. For these reasons, most liver injury biomarker research has been done using samples from mice or humans after APAP overdose. Unfortunately, few studies have been designed in such a way to permit calculation of positive and negative predictive values (PV). Nevertheless, nearly every study comparing a novel biomarker of liver injury with ALT and/or AST has found evidence that these new biomarkers perform better than the aminotransferases for detection of liver injury and prediction of outcome. The major categories of emerging biomarkers are summarized in Table 2(Tab. 2) and described below.

### Mitochondrial damage biomarkers

Mitochondrial damage and dysfunction are thought to be common mechanisms of drug hepatotoxicity (Pessayre et al., 2012[53]) and are especially important in APAP toxicity (Jaeschke et al., 2012[24]). Several biomarkers of mitochondrial damage have been identified or suggested. It has been shown that the mitochondrial enzyme glutamate dehydrogenase (GLDH) and mitochondrial DNA (mtDNA) are increased in circulation of humans after APAP overdose and hypoxic hepatitis, and that these markers are specific for injury involving mitochondrial damage (McGill et al., 2012[46]; McGill et al., 2014[47]; Weemhoff et al., 2016[68]). More recent research has identified acylcarnitines (McGill et al., 2014[45]; Bhattacharyya et al., 2014[8]), and the mitochondrial matrix enzymes carbamoyl phosphate synthetase-1 (CPS1) (Weerasinghe et al., 2014[69]; Brown et al., 2014[11]) and ornithine carbamoyltransferase (Furihata et al., 2016[19]) as possible biomarkers of mitochondrial dysfunction. Although all of these biomarkers appear to be useful for translational research (McGill et al., 2012[46]; 2014[47]), their clinical utility so far appears to be limited. Although several of them are higher in serum from non-survivors of APAP overdose than survivors, all those tested have relatively low sensitivity and specificity for patient outcome (e.g. 77 % and 76 %, respectively, for mtDNA at an optimal cutoff of 14 ng/mL) (McGill et al., 2014[47]). Nevertheless, they represent a first step toward development of biomarkers that predict outcome better than current liver injury tests (McGill et al., 2014[47]), as serum ALT levels show no association whatsoever with outcome (Björnsson et al., 2006[9]; Antoine et al., 2012[3]; McGill et al., 2014[47]).

### Cell death mode biomarkers 

The two major forms of cell death are oncotic necrosis and apoptosis, and biomarkers are available for the measurement of each. Currently, the most popular circulating biomarkers of cell death mode are the full-length and caspase-cleaved forms of keratin-18 (K18). Importantly, there is evidence that K18 is useful for prediction of patient outcome. Elevated serum levels of K18 at the time of presentation have been shown to predict later development of liver injury in early-presenting APAP overdose patients with positive PV of 64 % and 73 % for the full-length and apoptotic forms, respectively, and negative PV > 85 % (only 86.5 % for full-length K18), using optimal cutoffs (Antoine et al., 2013[2]). Although these values may not be good enough for these markers to serve as the major criteria in patient triage (because the antidote for APAP, N-acetylcysteine, is effective and relatively innocuous, it would be particularly bad to miss ~15 % of patients in need of treatment), they are much better than the values for ALT (negative and positive PV of 36 % and 85 %, respectively). Furthermore, significant associations between K18 and another cell death biomarker, high-mobility group box 1 protein (HMGB1), with poor outcome were observed (Antoine et al., 2012[3]). Interestingly, the greatest positive and negative PVs of any biomarker tested for early detection of liver injury after APAP overdose were for HMGB1, at 91 % and 87 % (Antoine et al., 2013[2]). Caspase activity and caspase cleavage can also be measured in circulation as markers of apoptotic cell death (McGill et al., 2012[46]; Woolbright et al., 2015[71]), but their clinical utility has not been assessed.

### DNA damage biomarkers

DNA damage can be assessed by laddering or smearing on an agarose gel, or by detection of nucleosomes by immunoassay. Nuclear DNA fragments have been measured using the latter approach in serum and plasma from patients with APAP-induced liver injury (McGill et al., 2012[46], 2014[47]) and acute liver injury due to other etiologies (Craig et al., 2011[12]; Weemhoff et al., 2016[68]) and are generally increased over healthy controls. They also appear to be higher in non-survivors compared with survivors (McGill et al., 2014[47]); however, sensitivity and specificity are lacking.

### Nucleic acid biomarkers

The relatively recent discovery that microRNAs (miRNA) can be detected in circulation has led to an explosion in interest in miRNA and other nucleic acids as non-invasive disease biomarkers (McGill and Jaeschke, 2015[44]). Multiple studies have reported increases in miR-122, miR-192, miR-125b and other miRNAs in serum or plasma after APAP overdose in both humans and mice (Ward et al., 2014[67]; Krauskopf et al., 2015[33]; Yang et al., 2015[77]; McGill and Jaeschke, 2015[44]), and some of these miRNA species appear to be very sensitive for drug hepatotoxicity in some cases (Dear et al., 2014[16]). Positive and negative PV of miR-122 for early detection of hepatotoxicity after APAP overdose were similar to K18 variants at 73 % and 87 %, respectively (Antoine et al., 2013[2]), and miR-122 is elevated in APAP-induced liver injury patients with poor outcome (Antoine et al., 2012[3]). Importantly, the use of miRNA biomarkers may go beyond detection of liver injury and prognosis in liver disease; circulating miRNA profiles may also be useful for diagnosis of the underlying cause of the injury. For example, circulating miRNA profiles have been shown to differentiate between APAP hepatotoxicity and hypoxic hepatitis (Ward et al., 2014[67]). 

### Other biomarkers

Changes in a variety of other serum and plasma biomarkers have recently been reported in liver injury patients. For example, argininosuccinate synthetase appears to increase earlier than ALT in acute liver injury and may be more sensitive (McGill et al., 2014[42]; Qin et al., 2016[56]). Certain bile acids are increased in acute liver injury (Woolbright et al., 2014[72]; Luo et al., 2014[40]). Even markers typically used to diagnose or follow conditions other than liver injury have been shown to be elevated in circulation of liver injury patients and can be useful for prediction of outcome, including kidney injury molecule-1 (KIM-1) (Antoine et al., 2015[4]) and troponin I (Moore et al., 2013[48]). Other proposed biomarkers include aldolase B (Qin et al., 2016[56]) and macrophage colony-stimulating factor 1 (Stutchfield et al., 2015[64]). Panels of biomarkers designed to maximize sensitivity and specificity have also been suggested (McGill et al., 2014[47]; Qin et al., 2016[56]). Finally, various cytokines and inflammatory markers have been shown to be elevated in circulation of patients with liver injury, including IL-6, IL-8 (James et al., 2005[25]) and acetylated HMGB1 (Antoine et al., 2012[3]; Weemhoff et al., 2016[68]).

## Conclusions

The purpose of this review is not to eulogize the aminotransferases, or any other markers of liver injury. Certainly, they will remain important tools for the diagnosis and study of liver injury in the future. Rather, the purpose is to provide the reader with a better understanding of the aminotransferases and to discuss emerging biomarkers that could replace or complement them in the future. It's clear that several novel liver injury biomarkers have better clinical performance than ALT and AST for early detection of liver injury and for prediction of outcome. However, better predictive values are desirable (improved NPV for patient triage and improved PPV for poor outcome after liver injury develops) before routine clinical use. The latter may be achieved in part through the use of multi-biomarker panels to maximize sensitivity and specificity, or through discovery of entirely new markers of liver injury. There is also evidence that certain markers, such as GLDH, mtDNA, nuclear DNA fragments, K18 and HMGB1 can indicate specific pathophysiological mechanisms, and this makes them useful for translational research. Finally, additional studies are needed to determine if any of these emerging biomarkers are useful for prediction of idiosyncratic toxicity in drug trials. 

## Acknowledgements

The author would like to thank the clinical chemistry fellowship program at Washington University School of Medicine for support, as well as Drs. Brian N. Finck and Manuel Gutiérrez-Aguilar at Washington University for helpful discussion. 

## Conflicts of interest

The author declares no financial conflicts of interest.

## Figures and Tables

**Table 1:**
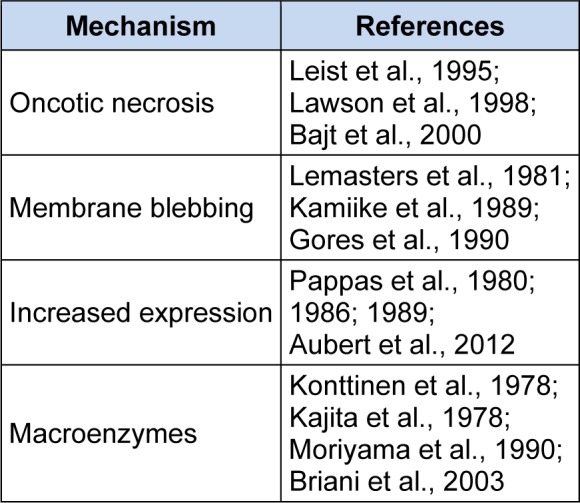
Mechanisms of plasma ALT or AST elevation

**Table 2:**
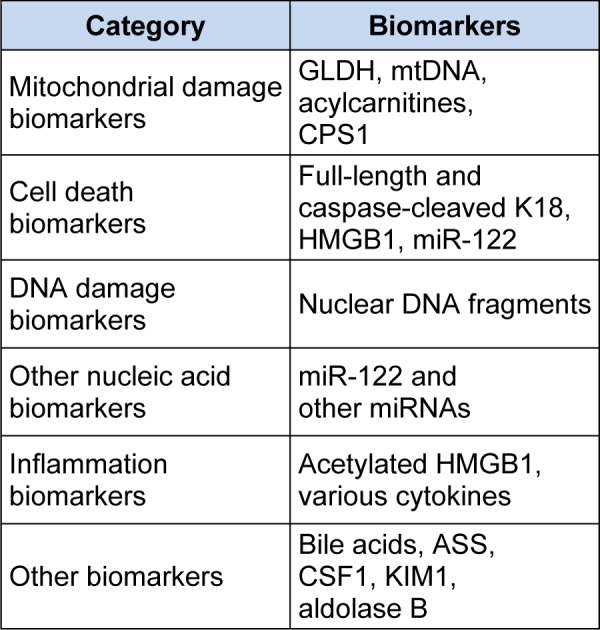
Examples of emerging liver injury biomarkers
